# Supplementary figures and images for: Signals of propaganda—Detecting and estimating political influences in information spread in social networks
Source: PLoS One. 2025 Jan 30;20(1):e0309688. doi: 10.1371/journal.pone.0309688 (PMC11781619; doi:10.1371/journal.pone.0309688)

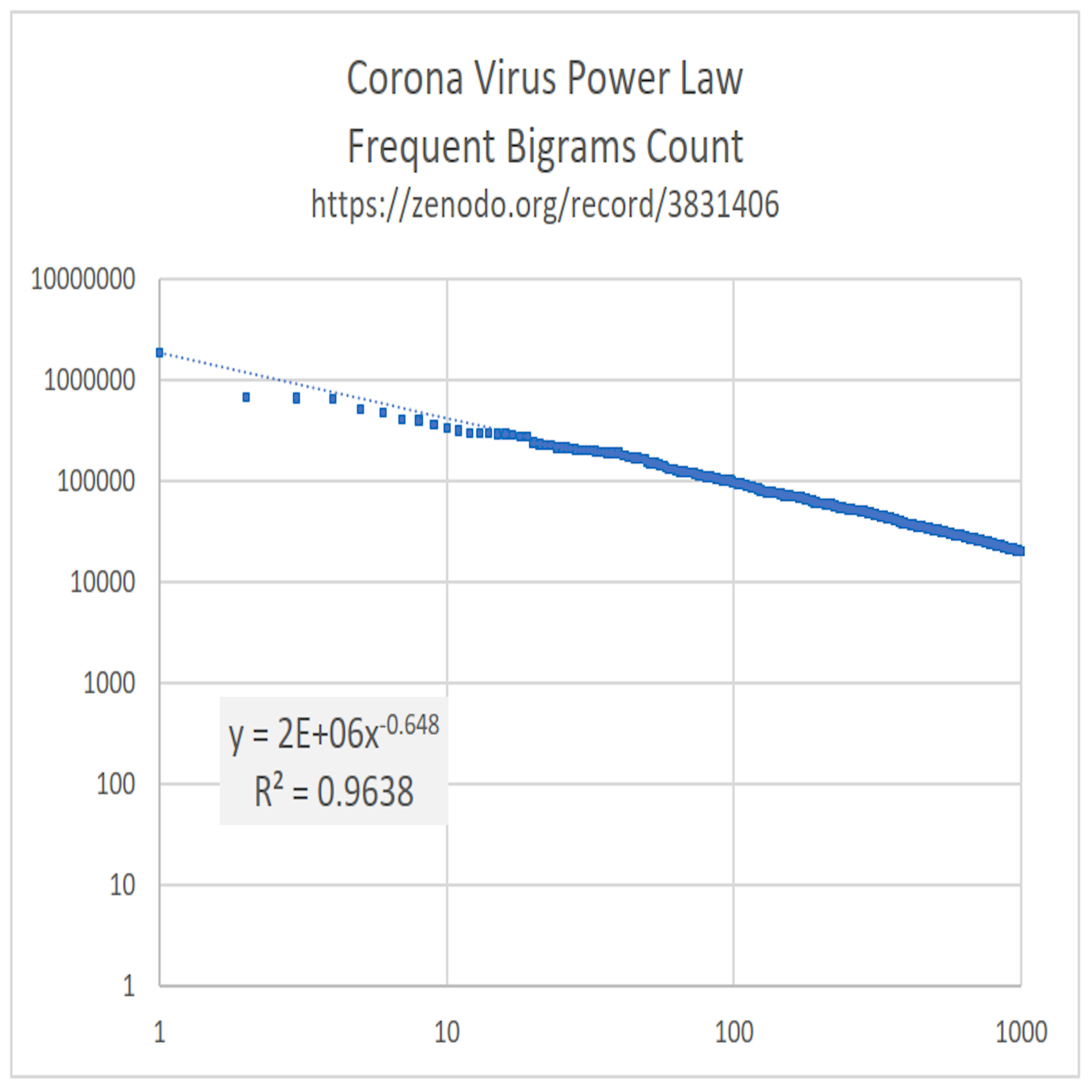

Supplement: S1 Fig — The slope is more similar to the political cascades than it is to the disaster cascades. (TIFF) [file pone.0309688.s007.tiff]

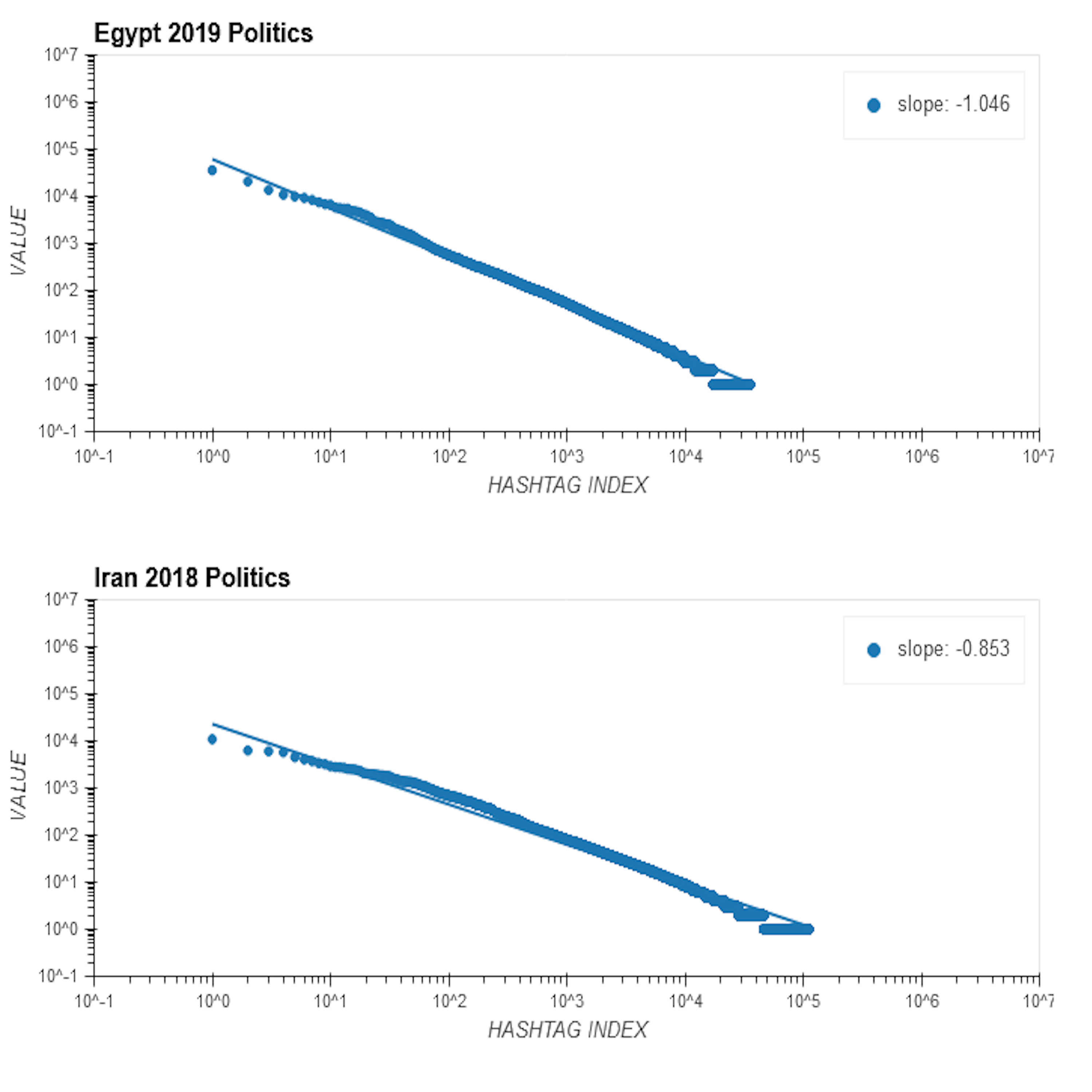

Supplement: S2 Fig — (TIFF) [file pone.0309688.s008.tiff]
