# Supplementary material for: Signals of propaganda—Detecting and estimating political influences in information spread in social networks
Source: PLoS One. 2025 Jan 30;20(1):e0309688. doi: 10.1371/journal.pone.0309688 (PMC11781619; doi:10.1371/journal.pone.0309688)
Supplement: Attachment — Submitted filename: Response to reviewers.pdf [file pone.0309688.s009.pdf]

Response to reviewers

Thank you for the time and effort to read and comment our manuscript ***"Signals of Propaganda: Detecting and Estimating Political Influences in Information Cascades in Social Networks".*** Thank you also for your beneficial comments, which we feel truly helped us improve our manuscript.

In our study, we evaluate how information cascades that include a non-organic spread, i.e., which is suspected to include some bias resulting from an effort to spread an agenda, and as such named "computational propaganda", can be detected on Twitter via their macro-scale network properties, without examining the text itself.

Our proposed detection method is based on the property of rhetorical speech (which is a part of propaganda) to include **a certain degree of repetition** in the messages i.e., "brainwash" or "slogans", and in the tendency of political discussions to repeat broadcasting similar messages to their target audience.

This creates a distribution where several terms repeat more times than in an "organic" discussion, for example, we use large scale disasters as a comparison group.

We show how such repetitions can be detected through their *Power law* exponents features, for both users and hashtags distribution. We show also how transforming of the cascades into bipartite graphs, connecting tweets and the words appearing in these tweets, can help find this type of repetitiveness when hashtags are lacking or in cases where hashtags are unevenly distributed between the political cascades and the comparison group.

As a comparison group to the political information cascades, we use information cascades related to *large-scale disasters*. We believe these cascades reflect a more "organic" cascade type since people in harsh situations share their knowledge and emotions for the sake of pure human cause, and not for the sake of spread or personal gain, and thus disasters cascades are less centralized in their nature.

Also, we developed a distinctive quantitive measure, named the *PLMSE measure*, based on our distinction above. We find that the political discussions tend to be more centralized, i.e., fewer users repeat similar words more often than it would have occurred in a n "organic" discussion. On the other hand, authentic social network conversations are more heterogeneous in terms of their used vocabulary.

Following the reviewer`s comments, we added an entire section explaining our PLMSE formula. We also added statistical tests to show the separation`s significance as long as many other corrections. We reorganized the GitHub repository entirely to enable a reproduceable work.

Thank you for this opportunity and your consideration of this manuscript as well as for the additional time that we received for its corrections and the deep remarks which improved the readability and clarity of our work.

Responses to the first reviewer are shown directly after this letter, while responds to the second reviewer starting at page 6. Additional comments and corrections following the editor's comments (which in some cases were similar to the reviewer`s comments) are allocated to the letter to the editor document and here starting at page 10. When the editor and the reviewer`s comments were on the same topic, we only answered the editor for to reduce redundancy.

We look forward to hearing back from you soon.

With kind regards,

Alon Sela (on behalf of the authors).

Reviewer 1

Comment: Reviewer #1: Sela et al. explores cascades of tweets relating to political topics and disasters, finding that for disasters the cascade distribution better follows a power-law whereas for political topics, the distribution falls off more quickly. The authors hypothesize about why this might be, noting various ideas from the literature. The paper is on an interesting topic and suggests an interesting comparison, along with novel findings.
At the same time, several points should be clarified before publication.

Answer: Thank you for this positive general evaluation.

Comment: On page 2 the authors suggest they use the disaster information as a control group for political cascades. I'm a bit concerned about this as each group has unique aspects and there are not other types of information explored beyond disasters. Perhaps suggesting this is just a comparison rather than a control is sufficient, or more carefully qualifying the idea of this as a control group. **Likewise, the comment on page 3 that disaster cascades are not likely to be manipulated is not supported and I am not sure that this is certainly true.**

Answer: Thank you for this comment. Indeed, following the reviewer's comments, we expressed our belief of a weaker degree of manipulation in disasters compared to politics **<u>in a more careful way</u>**.

> In the current study, we compare 22 data sets of large information cascades. These include 10 cascades related to political topics and 12 cascades related to non-political ones. We use as a comparison group for the political cascades, non-political data sets related to disasters. We believe that disasters related information cascades include lower direct gains from the manipulation of information and are therefore probably a good candidate for a comparison group to the political cascades. This belief is based on the following arguments:
>
> (1) Natural disasters-related information cascades might be less likely to be biased because a disaster or national emergency is experienced by larger portions of the society (less likely to be extremist or marginal parts of society). This applies even if the disaster is regional, e.g., 9/11 terror attacks, 2011 Fukushima nuclear disaster, 2013 Vanuatu tsunami, 2015 Nepal earthquake, 2017 Gulf coast hurricanes, etc. In these cases, many of the users expressing their opinions are "regular users" with no aim nor experience in methods to spread their message. They communicate in social networks simply to share, get informed, and help others.
>
> (2) [illegible]

Comment: The PLMSE measure is not fully clear to me. Conceptually I believe the authors are fitting a power-law to the data and then asking how far from the fit the data appears. However, Eq. 1 does not really appear like a MSE of a fit. Perhaps they could show the derivation more carefully (in SI) to explain better? Likewise, the authors describe 'n' as being the number of points, yet it seems to me that this might be the number of bins in the power-law distribution? Otherwise, how do they have an expectation for what each point should be (since they're drawn from a distribution)?

Answer: Thank you for this important comment. We added to the article an explanation of the PLMSE formula. Indeed, the PLMSE formula was not clear, and we hope now it is clearer.

Explaining the PLMSE formula

The following section explains the PLMSE formula and each of its components. Overall, Equation 1 has 3 main components: (1) $\frac{n+1}{n}$, (2) $\frac{1}{i\cdot(i+1)}$ and (3) $\log^2(\frac{y_i}{c}\cdot i^{\alpha})$. Let us start from part (3). We denote i as the index of the bin (x-axis) and similarly y_i is the frequency of the distribution at point i, in our case the y-axis. To compute a simple MSE, on a linear line, we need to compute the expression $\frac{1}{n}\cdot\sum_{x=1}^{n}(y_x-(ax+b))^2$ where a and b are the linear line coefficients. In our case, where our data is not linear, but assuming it is a power law distribution, then it should be linear on a log-log scale.

We thus transform:

$$\overline{x}: x \rightarrow log(x)$$
$$\overline{y_x}: y_x \rightarrow log(y_x)$$
$$line: y'_{\overline{x}} = -\alpha\cdot\overline{x} + log(c)$$

We now define the squared error in terms of the Log-Log line:

$$\sum_{i=1}^{n} w_i\cdot(\overline{y_i}-y'_i)^2 \rightarrow \sum_{i=1}^{n} w_i\cdot(log(y_i)-(-\alpha\cdot log(i)+log(c)))^2$$
$$\sum_{i=1}^{n} w_i\cdot(log(y_i)-(-\alpha\cdot log(i)+log(c)))^2 = \sum_{i=1}^{n} w_i\cdot(log(y_i)-(-\alpha\cdot log(i)+log(c)))^2$$
$$= \sum_{i=1}^{n} w_i\cdot(log(y_i)-log(c)+(log(i^{\alpha}))^2$$
$$= \sum_{i=1}^{n} w_i\cdot(log(\tfrac{y_i}{c})+(log(i^{\alpha}))^2 = \sum_{i=1}^{n} w_i\cdot log^2(\tfrac{y_i}{c}\cdot i^{\alpha})$$

As for part (2) of the equation, we constructed modified weights that give a higher focus (weight) on the upper left data points in the straight line. This uneven weighting system is needed since in the Power Law distribution, these points represent many of the observation, while the lower right parts (the long tail) represent only a few. Thus, to give a fair representation as required to compute the slope properly, and to construct a proper weights, first notice that the following equation holds:

$$\sum_{i=1}^{n}\frac{1}{i\cdot(i+1)} = \sum_{i=1}^{n}(\frac{1}{i}-\frac{1}{i+1}) = (\frac{1}{1}-\frac{1}{2})+(\frac{1}{2}-\frac{1}{3})+...+(\frac{1}{n}-\frac{1}{n+1}) =$$

$$1-\frac{1}{n+1} = \frac{n}{n+1}.$$

Then, as for part (1) of the equation, we need this part such that the normalized weights in part (1) and their multiplication by part (2)

$$\sum_{i=1}^{n} w_i = \frac{n+1}{n}\cdot\sum_{i=1}^{n}\frac{1}{i\cdot(i+1)} = 1$$

of the PLMSE equation, are **always** equal to 1.

8

Comment: In Figure 3, the authors don't show the US elections it seems. Is there a reason for this? Are the results similar for the US as the others? Likewise, the 'user_index' label is unclear to me. Is this just the number of users that e.g., have 1 tweet, 10 tweets, etc. on the topic?

Answer: Thank you for this comment. In figure 3 we show 6 vs 6 politics and disasters, and in fig 4 we show the entire data**.** We simply showed part of the data as histograms due to space limits. We added to the git the additional images from which we compute the slopes which are the values that separate between the numbers. As for the user_index, indeed it is the number of user when sorted from the most to the least frequently appearing user in the data.

Comment: The caption for this and Fig. 2 should be more detailed.

Answer: Thank you for this comment. We added to the caption the following explanation.

> **Fig 2.** Distribution of hashtags on a log-log scale for 6 political (left column) vs. 6 disasters (right column) cascades. We only show here 6 disaster and political cascades distributions, while the scores and PLMSE scores for all the data are shown in Fig 4(C-D). Mean slopes are -0.938 / -1.214 for the political/disasters cascades, a significant difference (t-test, p-value = 0.0041). For the political cascades, most slopes values $\alpha < -1$, while for the disasters cascades, all $\alpha < -1$. When using all 10 political cascades (and not only 6) this significance even grows (t-test, p-value = 0.00187).

Comments: The authors claim that the separation in Fig. 4 is clear between disasters and politics seems a bit overstated. Perhaps the authors could provide a metric on how good their method is at making predictions? Could they maybe calculate an AUROC based on the scores or give another standard metric? Eyeballing, the graph for example in 4D many points in politics are comparable to disasters (e.g., USA politics 2011).

Answers: Thank you for this important comment. We added a t-test and show the p-values which were computed and added to the manuscript in the caption of Fig.4.

> **Fig 4.** Separation between political (orange) and disasters (blue) cascades. (A) slope of user distribution, (B) PLMSE scores of user distribution, (C) slope of hashtags distribution, (D) PLMSE scores of hashtags distribution. Note the slope difference for the users and the hashtags and the differences between the political and disaster cascades. Means of political and non-political cascades are found significantly different. T-test; p-value = 4.11E-5 / 0.00045 / 0.00187 / 0.0026 for sub-images (A)-(C) respectively.

Comments: repetitive broadcasting' should be 'includes repetitive broadcasting'; on page 2 'of a political discussions' should be 'of political discussions'; on page 5, 'would be explain' should be 'will be explained'; also on page 5 'the researcher study' should be 'the researcher studies'; also on page 5 'get Power law' should be 'get a power law'; on page 6 'data sets where used' should be 'data sets were used'; also on page 6 'scarping' should be 'scraping' and 'scraps' should be 'scrapes'; on page 8 'twits' should be 'tweets'; on page 14 'additianl' should be 'additional'.

Answer: Thank you and our apologies for this issue. We thoroughly spell check the article again. We believe that now all spelling mistakes have been corrected.

Comments: On page 2 the statement that bots are illegal might be overstated, rather I think it is just a violation of terms of service potentially.

Answer: Thank you for this comment, we rewrote the sentence in a more subtle manner to correctly describe the reality where bots in many cases are legal (see below):

> Ben-Gal, 2019; Vasilkova & Legostaeva, 2019). In addition to bots (which some of them are illegal since they pretend to be real humans), information spreaders might use legal methods to increase the spread of a political agenda and increase one's influence include the use of opinion leaders, or social media management platforms (Sela, Cohen-Milo, Kagan, Zwilling, & Ben-Gal, 2020; Sela et al., 2019). One should note

Reviewer 2

Comment: The paper proposes the analysis of several datasets of Twitter posts to evaluate the statistical differences between political and non-political discussions.
The proposed evaluation starts from the hypothesis that political discussions (as propaganda) generally leverage the repetition of messages and involve a limited number of users when compared with non-political discussions. The authors are able to distinguish between political and non-political datasets by studying the properties of graphs generated with the different datasets. Moreover, the authors propose the construction of a bipartite graph in which each tweet is connected with the words it contains.
The results emerge by studying how closely the graph properties exhibit theoretical power-law distributions and focusing on the evaluated parameters to derive the slope and an additional score called PLMSE (power-law mean square error).
Strong points:
- an attractive and reasonable approach
- a pleasant writing style

Answer: Thank you very much for this positive evaluation. We will do our best to address the weak points.

Comment:

Weak points:
- not convincing experiments
- lacking of crucial details
- limited number of tests
- detection motivation not fully plausible
- typos

Answer: Thank you for this evaluation. We added t-tests to statistically support our claim that the political and nonpolitical cascades differ. We did not perform experiments, but rather analyzed large datasets where some include millions of tweets. All together, we analyzed networks with over 19 million edges and 3 million nodes. For the analysis that did not include the full cascade network, we analyze almost 24 million records, of which we concluded these results.

This is not a negligible data set, and each cascade topic can be of many millions' records. The full data sets sizes and records used for the network analysis and non-network analysis are found in the Git of the project in a file named "DataSources.xlsx" and Network_datasets.csv". We corrected the comments and concerns raised by the reviewers.

We added the statistical significance to estimate the difference between the attributes of the political /non-political cascades and added also a mathematical explanation to our PLMSE method of numerical estimation of the cascade`s type.

Typos have been corrected.

Comment: The bipartite graph-based approach is attractive and sounds relevant with respect to the traditional NLP-based ones. However, while the paper's approach is attractive, its overall quality and impact seem limited, and the results appear not entirely sound. First of all, many important details appear to be overlooked when describing the evaluation methodology. For example, it is unclear how the authors processed the tweets and which words were included in the graphs (what about the stop words and alike?) or which are the details of the operation.

Answer: Thank you for this comment. We added the following paragraph to the article to clearly explain this process.

> To construct a bipartite graph from each cascade, we started by a conventional stemming and lemmatization processes that removed common words such as "and / or / if. . . etc.." from the tweet text. These were done by the NLTK python package. Then, we constructed a link between tweets and words appearing in these tweets connecting words appearing in each tweet to create a weighted link between words and their tweets for each tweet, where the weight is according to the number of times similar words appear together. Figure 1) illustrates this process. These links form a graph for each cascade, connecting words to tweets. These graphs were constructed both for the political and for the non-political topics. We computed several graph

Comment: "The data was binned prior to the slope estimation". The plots reported in the figures are inconsistent and incomplete: it is unclear why some datasets are used for showing some properties but not for others (for example, USA-2016 and Catalonia-2006 are missing in Figure 3).

Answer: Thank you for this comment. Your observation that *"some datasets are used for showing some properties but not for others"* in Figure 3 is correct. Figs 2 and 3 show each 6 data sets of political and 6 of nonpolitical cascades. Figs 4 and 5 in comparison, show 6 disaster cascades but 10 points (and not 6) related to political cascades. The reason why we show in Fig 2 and 3 only 6 X 2 images is simply for ease of presentation space and since we only found 6 disaster's datasets of large enough size.
Furthermore, based on figs. 2,3, one cannot truly conclude that these images are different. It is rather more of a demonstration that illustrates the phenomena and the deviation, mainly on the upper left side in the political cascades. **The images show the conceptual idea but does not quantify it.** This quantification is shown in fig 4 and 5 in contrast, where each cascade is only marked by a single point (and not as an entire graph). Thus, we can show all data sets that were available to us, which are 10 political cascades and 6 disaster cascades.
Following the reviewer`s comment, we corrected the caption of images 2 and 3 and emphasized that this is only a part of the datasets.
In addition, we add to the SI section a table containing the data points for all 10 political points and 6 disaster points. Following the reviewer's comments we added this paragraph

> **Figure 2.** Distribution of hashtags on a log-log scale for 6 political (left column) vs. 6 disaster (right column) cascades. Note that since we only collect 6 disasters cascades datasets, we show here in accordance 6 (out of 10) available political cascades. We compute the slopes of the additional 4 cascades and present them in Figure 4(C). Mean exponents (slopes) are -0.804 and -1.247 for the political topics / disasters topics distributions respectively, and are significantly different (t-test, p-value = $9.7e^{-5}$) on this sample of 12 cascades

Comment: Another questionable point is the claim on page 7, where considering "words used in the tweet regardless if they are hashtags or not" would remove the bias that "political users might tend to use hashtags more often than regular users". This way, political discussions will include the words related to the tweet *and* the hashtags downgraded to regular words.

Answer: Thank you for this correct comment. When analyzing the tweets, we removed first stop words. We performed a standard lemmatization which include this work removal. Thus, we do not include in our analysis of bipartite graphs words such as and / or / etc. in the tweet. We used the NLTQ and scikit-learn python packages that do this already.

Comment: The use of disaster-related datasets is a reasonable sample. However, it is only one of the possible types of "cascades" found on Twitter. How do the results differ when considering discussions about other events, like the Super Bowl, lengthy expected movies, or World Cup exhibitions?

Answer: Thank you for this comment. We understand the point of comparing additional cascades to the 16 available cascades. We added the Covid19 frequent bigrams cascades to the SI section, and saw that according to its slope, Covid 19 cascades are more similar to a political issue than it resembles a disaster. This is since its slope is -0.648. We plan to perform such an analysis on the coming USA election, but this analysis is out of the scope of the current work.

Comment: Furthermore, motivating the proposed methodology to detect political discussion sounds relatively trivial, considering that the presence of given hashtags proves that a tweet is political.

Answer: Thank you for this comment. Truly finding political topics is trivial. Our method does not offer only method to find pollical discussions (which clearly does not much effort), but rather finds interventions in a discussion that are not “organic” in the sense that the distributions deviates from the organic discussion. We use politics since we know that politics has a clear goal – to push an agenda. But in other cases, and topics this is not clear. For example, in health-related topics, it is not always clear if it’s an organic discussion or not. Furthermore, if we do not know in advance if a discussion is political or not, for example Global Warming or Immigration, our method permits us to determine for any given topic if its more organic (authentic users discussion) or more politic (specific key holder that aim at spreading their agenda. One should note that we did not inspect the textual meaning or content of the hashtag itself, nor if it was a hashtag or a simple word. We only look at the distributions and their slopes parameters.
The method used does not pretend to distinguish solely political propaganda, but mainly an amplification of some words, which appears in political messages but also might appear in commercial communication.
Indeed, we mention in the introduction on page 2 that “*Pre-election political messages tend to be more aggressive, populistic and in general, use similar techniques as the ones used in commercial communication. These commercial communication techniques repetitively broadcast a few well-defined messages, to penetrate the minds of the exposed audience and affect the memory recall for a product or similarly, a political player.”*

We therefore see political cascades as a special case of twitter discussion where users have a clear goal, to push an idea or an agenda. This differs from other conversations, where the users simply publish their own opinions. Following this remark, we added the following correction to the manuscript.

> Zwilling, & Ben-Gal, 2020; Sela et al., 2019). One should note however that we use in this study the term "propaganda" in the context of an effort to change public's opinion. While the initial use of this term has been mainly associated with totalitarian regimes, it has also been used for many years, in softer regions of marketing McGarry (1958). We use here this term in its wider and contemporary context Hobbs (2020), which defines propaganda as tools that include *"filtered digital content, targeted advertising, and differential product pricing to online users"*. We follow this line and use propaganda [illegible]

Comment: Finally, several typos undermine the overall quality of the paper: it seems that the paper did not receive a last text revision. A prominent example is the word "style" on page 11, showing that a spell-check was missing before the submission to a prominent venue like PONE.

Answer: Thank you and our deep apology for this mistake. We spelled-checked the entire article again. We are very sorry for these mistakes.

Comment: [NOTE: If reviewer comments were submitted as an attachment file, they will be attached to this email and accessible via the submission site. Please log into your account, locate the manuscript record, and check for the action link "View Attachments". If this link does not appear, there are no attachment files.]
While revising your submission, please upload your figure files to the Preflight Analysis and Conversion Engine (PACE) digital diagnostic tool, https://pacev2.apexcovantage.com/. PACE helps ensure that figures meet PLOS requirements. To use PACE, you must first register as a user. Registration is free. Then, login and navigate to the UPLOAD tab, where you will find detailed instructions on how to use the tool. If you encounter any issues or have any questions when using PACE, please email PLOS at figures@plos.org. Please note that Supporting Information files do not need this step.

Answer: Thank you again for the helpful comments. We made every effort to answer and correct the manuscript so that these comments are addressed and corrected. We wish to thank the editor and the reviewers again for the time and effort they have spent to improve our manuscript.

Additional comments based on Editor` s comments.

1. General comments and comments by the editor:

Comment: Please ensure that your manuscript meets PLOS ONE's style requirements, including those for file naming. The PLOS ONE style templates can be found at https://journals.plos.org/plosone/s/file?id=wjVg/PLOSOne_formatting_sample_main_body.pdf and https://journals.plos.org/plosone/s/file?id=ba62/PLOSOne_formatting_sample_title_authors_affiliations.pdf

Answer: We have ensured the style meets PLOSONE requirements.

Comment: In your Methods section, please include additional information about your dataset and ensure that you have included a statement specifying whether the collection and analysis method complied with the terms and conditions for the source of the data.

Answer: The entire data sets were collected from open sources. All links for the datasets and the code used to analyze the data were uploaded to the project`s **GitHub repository**, which is now open to public and are available at OmerNeter/tweets_politics_project (github.com) The full link of the git is: https://github.com/OmerNeter/tweets_politics_project

In this GitHub repository, we added a table named “DataSources.xts”, where the attributes for all the data sets including their size and links are shown. All data sets come from open access data repository, and thus there are no restrictions of sharing their links (but they cannot be uploaded to our GitHub but due to their size and ownership). We also added an example (one or more) to the repository, to enable an easy run of the code and for making the code reproducible. The GitHub is organized in 3 sub folders, “users”, “hashtags”, “network” similar to the 3 types of analysis presented in the article.

Comment: Please note that PLOS ONE has specific guidelines on code sharing for submissions in which author-generated code underpins the findings in the manuscript. In these cases, all author-generated code must be made available without restrictions upon publication of the work. Please review our guidelines at https://journals.plos.org/plosone/s/materials-and-software-sharing#loc-sharing-code and ensure that your code is shared in a way that follows best practice and facilitates reproducibility and reuse.

Answer: Thank you for this remark. All code or links to the data which were downloaded from different repositories are found in the GitHub repository of the article. It is a public repository which can be access by all. https://github.com/OmerNeter/tweets_politics_project

We added to the root of the GitHub a file named “DataSources.xlsx” which contains the types of data sources, and in column J the link for downloading it.

Comment: Please note that funding information should not appear in any section or other areas of your manuscript. We will only publish funding information present in the Funding

Statement section of the online submission form. Please remove any funding-related text from the manuscript.

Answer: Done!

Comment: We note that the grant information you provided in the 'Funding Information' and 'Financial Disclosure' sections does not match.

Answer: Thank you for this comment. We removed any funding information from the article itself and only kept the funding acknowledgment in the editorial manager.

Comment: Thank you for stating the following financial disclosure: "no". Please state what role the funders took in the study. If the funders had no role, please state: "The funders had no role in study design, data collection and analysis, decision to publish, or preparation of the manuscript."

If this statement is not correct you must amend it as needed.
Please include this amended Role of Funder statement in your cover letter; we will change the online submission form on your behalf.

Answer: We uploaded a new cover letter where we added a declaration that "**The funders had no role in study design, data collection, and analysis, the decision to publish, or preparation of the manuscript"**.

Comment: In your Data Availability statement, you have not specified where the minimal data set underlying the results described in your manuscript can be found. PLOS defines a study's minimal data set as the underlying data used to reach the conclusions drawn in the manuscript and any additional data required to replicate the reported study findings in their entirety. All PLOS journals require that the minimal data set be made fully available. For more information about our data policy, please see http://journals.plos.org/plosone/s/data-availability.

Answer: The data points that were used for creating the charts were uploaded to the GitHub repository. We cannot provide the entire data since we are not its owner but rather several open sources repositories. In addition, all link to download ALL the data that are the base for reproducing the work were also uploaded to the GitHub repository of the project.

The URL for the GitHub repository (set to Public) has been added to the manuscript and is given below: https://github.com/OmerNeter/tweets_politics_project

As such, the manuscript satisfies the "minimal data" requirements as we comprehend it. "Upon re-submitting your revised manuscript, please upload your study's minimal underlying data set as either Supporting Information files or to a stable, public repository and include the relevant URLs, DOIs, or accession numbers within your revised cover letter. For a list of acceptable repositories, please see http://journals.plos.org/plosone/s/data-availability#loc-recommended-repositories. Any potentially identifying patient information must be fully anonymized.

Answer: We note that GitHub is in the list of approved repositories for PlosOne https://plos.org/open-science/open-data/#accessible-data

Also, therefore uploaded all the data points that were used to create the images to the GitHub repository, and they can be found at the root of the repository.

Commet: Important: If there are ethical or legal restrictions to sharing your data publicly, please explain these restrictions in detail. Please see our guidelines for more information on what we consider unacceptable restrictions to publicly sharing data: http://journals.plos.org/plosone/s/data-availability#loc-unacceptable-data-access-restrictions. Note that it is not acceptable for the authors to be the sole named individuals responsible for ensuring data access. We will update your Data Availability statement to reflect the information you provide in your cover letter.

Answer: The entire datasets used in this study were downloaded from existing data sources, (Kaggle, Figshare and digital library). There are no ethical issues in sharing the links to these datasets, but we cannot upload all the datasets to our GitHub repository because of two reasons. First, we do not own it, but rather, second, it is too large.

we share the links to downloading all the datasets used here in our GitHub repository. These are found in the excel table in the GitHub main page.

We also added to the GitHub processed datasets, such as network structures in .graphml files, and several datasets that are used to run the code. Mainly, we did not upload the entire datasets to the git but rather of several examples of cascades for each analysis.

Comment: We note that you have stated that you will provide repository information for your data at acceptance. Should your manuscript be accepted for publication, we will hold it until you provide the relevant accession numbers or DOIs necessary to access your data. If you wish to make changes to your Data Availability statement, please describe these changes in your cover letter and we will update your Data Availability statement to reflect the information you provide.

Answer: All code and several datasets to run the code are available in the GitHub repository of the project. The code was verified by an additional programmer other than its developer to verify it is working and has no issues of dysfunctionalities. **The links to the additional datasets (the entire data size was over 200 MB) can be downloaded from the links in the root directory of the git.**

Comment: Additional Editor Comments:
Please look at the detailed review by the reviewers. In addition to their helpful response, I would like to add the following review:
a. Propaganda is based on "partial truth or outright fiction" (page 2). Since you did not analyze the tweets' content, you cannot classify the tweets as true or not; hence, you actually measure and analyze the cascading of information. I think you should consider re-framing the paper as cascading rather than Propaganda.

Answer: Thank you for this comment. While the term propaganda is commonly used in the context of Russian or Fascist regimes, it also has a more recent and rather old definition, which goes beyond the definition of good (USA) and bad (Communist Russia). We use the definitions of recent studies and adopt here the term propaganda in its wider context. We add to the manuscript the following text explaining this issue.

> of opinion leaders, or social media management platforms (Sela, Cohen-Milo, Kagan, Zwilling, & Ben-Gal, 2020; Sela et al., 2019). One should note however that we use in this study the term "propaganda" in the context of an effort to change public's opinion. While the initial use of this term has been mainly associated with totalitarian regimes, it has also been used for many years, in softer regions of marketing McGarry (1958). We use here this term in its wider and contemporary context Hobbs (2020), which defines propaganda as tools that include *"filtered digital content, targeted advertising, and differential product pricing to online users"*. We follow this line and use propaganda as any intended effort to push a message or an agenda, to large audiences as possible, as oppose to simply publishing the message on the web and having others comment and discuss the content of the message through a natural discussion. This wide definition enables us to move from the negative association of "bad Russia vs. Good USA" to a more quantitative and neutral definition which includes any intervention in the natural process of information spread. In this aspect, any centralised effort to change opinion of masses is to some degree propaganda, even when its cause are good and true.

Comment: The example of tree height as power-law distribution should be mentioned in the citations you cite. The citations research the connection between tree diameter and its height. Actually, Barabasi and Albert discussed that height cannot be power-law distributed, as with such a case, we anticipate finding trees with 1 km of height.

Answer: We changed the example to city sizes which are known to be distributed by a Power law and have positive feedback of their sizes.

> [illegible]
>
> In some cases, however, this assumption does not hold. For example, if the researcher studies the size of cities, the growth of one city might not be independent of its current size. A large city attracts more people than a smaller one, thus city growth are not independent, but rather are dependent on their sizes. They will thus not be distributed according to a Normal distribution.

Comment: Methodology – Please describe the criterion for collecting the data for each dataset. If data is collected by their hashtag, that might explain the PL distribution.

Answer: We used existing repositories such that the data is open to all. We first analyze 6 political cascades from Kaggle. We then found 6 cascades related to disasters from the Digital Library repository. These data sets had already the aggregated counts of Users and Hashtags. We also collected the entire tweet message from similar repositories to create the bipartite graphs. For the creation of the bipartite network, we use again Kaggle for the political cascades and Fishgare data repository for the Disaster cascade. Since the data only included the tweet ID, we used a free scaping tool name Hydrator (https://github.com/DocNow/hydrator) which collects the tween text from the tweet ID. This enabled us to create a bipartite graph, which contains all the words and not only the Hashtags. Since we were concern that political actors use more often hashtags than regular users, we wanted to inspect the appearance of each word and not only hashtags, thus not to have a bias in the analysis. We could not control the method that the data was collected, but we did

use several repositories, of which some had only the aggregated data and other the full tweets to reduce to minimum the chances for such a bias due to sampling differences.

Comment: How is the data binned into bins?

Answer: We thank the reviewer for this comment. The data was binned by a logarithmic binning which is the common method for binning in case of long tailed distributions. We emphasized this point in the manuscript.

> **RESULTS**
>
> We show first the slopes of each of the 12 distributions - for the six political cascades and the six disasters cascades. We use the maximum likelihood method (Bauke, 2007) to find the slope on a log-log scale, after binning the data on a logarithmic scale, and compared the exponent parameter α of the Power Law distribution $y(x) = e^{-\alpha x}$

> ***The Data***
>
> We used three different data repository and over 13 GB of data to analyze the difference between the political and disasters cascades. First, we downloaded 6 data sets related to political information cascades from Kaggle (Mooney, 2019), and 6 other datasets related to disasters from the Digital Library Repository (UNT, 2021). The sizes of these data sets are shown in Table A1 and A2 in the SI section. These data sets where used to compute the slopes of the distributions, reflecting the speed of decay in each field and compare between these macro scale properties of political and disasters events. The distribution of users / hashtags on a log-log scale and the computed slopes of these datasets are presented in Fig. 2 and Fig. 3 respectively. The data was binned on a logaritmic scale prior to the slope estimation in order to eliminate the long-tail

Comment: . On page 7, the authors describe the network analysis they conducted. The results of this analysis should have been reported—for example, betweenness centrality, GC, and more.

Answer: Thank you for this comment. We reported only the features that made a significant separator -- that were relevant. **We added to the Github repository a new table named "Network_datasets.csv" which includes the main network properties** - *average degree, number of clusters, number of nodes*, *number of edges* and *slope on a log log scale*. We did not compute betweenness centrality since it is a node attribute and not a network attribute and also not the mean betweenness centrality since it's based on the betweenness centrality which was not calculated initially.

Comment: You mention the outlined frequency of the name of the disease hashtag (page 8). Since regression is sensitive to outliners, how will the results be changed if you omit this point?

Answer: We thank the reviewer for this comment which made us understand the issue is not clear. We modified the paragraph as seen below.

1999). In the case of the disasters cascades, we sometimes observe one single point in the histogram that appear above the line. After inspecting this point, we found that it is the name of the disaster event itself, e.g. *#florence*, *#dorian*, *#harvey*,...,etc which appears in all the twits. In contrast, in the political cascades we observe (left column of Fig. 2) several points above the line, and a weaker fit to the power law slope. These numerous points above the curve fit line, suggest that several of the mostly repeated hashtags are more common than would have been expected in politics. It is not simply one single hashtag that appears in all the tweets, but rather a set of several hashtags that are related to the political campaign cascade and those are repeating continuously. Examples of such political terms that are commonly spread in politics [illegible]

Regarding the outliers, since we made a logarithmic binning, the values of the log when doing a linear regression are less extreme. Thus, removing this point in the regression when in log scale would not change the slope much. Furthermore, although regression is sensitive to outliers, looking at the exponent is a very common method in Complex Networks. Indeed, one can see in fig 2 and 3 that the slopes were constructed upon many points, and therefore removing one single point will not change the slope dramatically. This is in fact one reason why we added the PLMSE measure, which gives greater weights to the most commonly appearing hashtag / user but still the slope is kept as an important factor. We thank again the reviewer for putting this unclarity into our attention.

Comment: Did you check the t-test assumptions? What flavor of t-test has been used? If you use two groups unequal variance test, the p-value significance is marginally

Answer: We thank the reviewer for this comment. We calculated the t-test and added top the manuscript. In page 10.

political cascades is $\bar{\alpha} = -0.93$ while for the disasters cascades, they are $\bar{\alpha} = -1.21$. These values differ significantly (t-test, p-value = 0.004) The sharper slopes in the power law distribution of the disaster cascades (i.e., larger absolute values of the neg-

….

cascades more than they should appear naturally, suggesting higher repetitions levels than we would expect. More importantly, the difference between the slopes of the two types of cascades is significant (t-test, p-value = 0.002) for 10 the political cascades compared to the 6 disaster cascades respectively, and it is (t-test, p-value = 0.004) when only 6 political cascades are used as in Figure 2, suggesting a real difference between the two types of cascades.

And for the users in fig 3 the t-test values as shown below.

Also, in the disasters cascades, we observe slopes that are substantially less sharp (slower decay) compared to the political cascades, i.e., (1.32−3.09) vs (0.572−0.82) for the political / non-political cascades respectively, which is statistically significant (t-test, p-value = 9.9E-5) and indicating a sharper loss of interest in politics discussions which can be understood by the sometimes negative emotional aspect of politics, - *when talking about politics, one either likes to talks about it, or does not talks about politics*

Comment: The data series for hashtags and users and the network data series differ. It seems like a cherry-picking series. It is better to combine all the datasets into one set so that you can perform all your research on them.

Answer: The data in Fig 2 / 3 and in fig 4 are mainly examples of the slopes. They differ in 2 data sets, where USA 2016 and Catalonia 2006 appear in the Hashtags (Fig 2), but instead of these datasets, in the Users figure (Fig 3) appear Egypt and USA 2011. In contrast, the aggregated figure (Fig 4) shows the slopes of all the 10 political data sets. These 10 political and 6 disaster cascades are not cherry picking, since one can see the differences in the slopes for all 10 political samples. We added Egypt 2019, USA 2011, and Iran to fig 4, mainly because we could not show 10 illustrative images in one page due to space limits in these images.

The good separation between all 10 political datasets compare to all 6 disaster data sets can be observed in Fig 4. This figure only shows the statistical properties of the cascade exponent (slope) and not the bipartite graphs attributes. Also, the t-tests between the 6 vs 6 data sets (fig 2 and 3) / 10 vs 6 data sets (fig 4) were performed, with p-values being 9.9E-5 / 4.1 E-5 for the 6 / 10 political cascades respectively of the users distributions. For the hashtag distributions, the t-tests p-values are 0.0018 / 0.004 for 6 vs 6 / 10 vs 6 t-tests respectively.

Additionally, following the reviewer`s comment, we added the missing image for the slope of Egypt and Iran Hashtags that did not appear in the previous version to the SI section (as can be seen below).

Comment: Have you considered that the politics users' distribution is not a power law?

Answer: Thank you for this comment. There is a big discussion (Clauset A et al) about the appearance of long tailed distribution vs Lognormal or other long tailed distributions or Power law distribution. In fact, the users are not a power law, and if so, then a truncated power law. As for the Hashtags, we base our understanding on previous work (*Zhou, B., Pei, S., Muchnik, L., Meng, X., Xu, X., Sela, A., ... & Stanley, H. E. (2020). Realistic modelling of information spread using peer-to-peer diffusion patterns. Nature Human Behaviour, 4(11), 1198-1207.*) where we show this distribution in more details. With this, we found much discussion in the literature about this question, where all agree about this distribution to be a fat tail, but several other distributions are also possibilities.

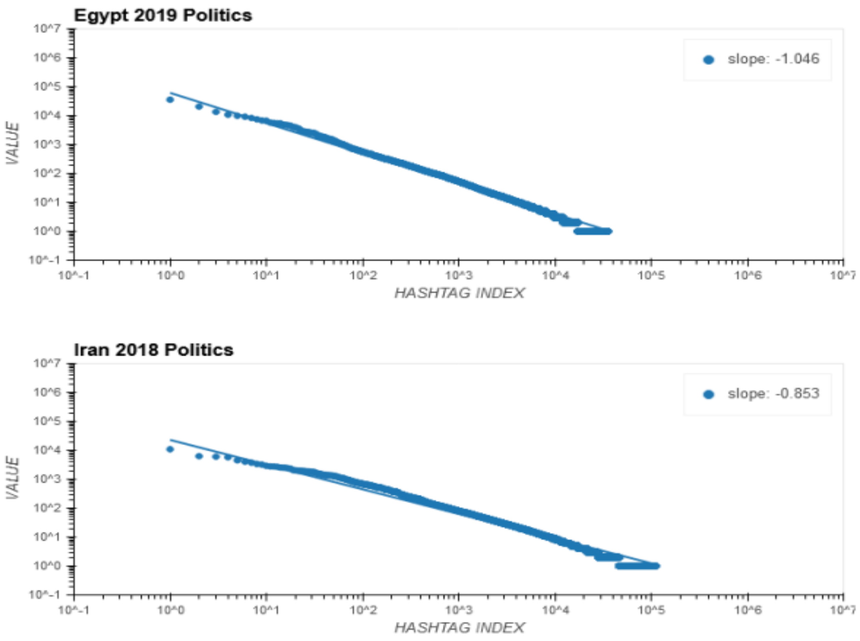

Figure A2. Additional slopes images (complementary to Fig 2 and Fig 3 for hashtags of Egypt and Iran politics.

Since we show in this article how the slopes can be used to separate between the political and the non-political cascades, then if the distributions are fully a "clean" power law or some fat tailed distribution that resembles a power law is not the main issue as long as we show that it is a fat tail distribution. We based our separation on the idea that more hashtags / users being hyperactive in the political compared to the disaster's campaigns, which is a part of an enhanced effort to spread an agenda, which we name propaganda. Based on the tendency of propaganda to include repetition, we think it is not critical if the slope is indeed a clean power law or just a long tail distribution.

The reviewer`s comment is important for the users' distributions, where it is rather clear that this is not a power law but a truncated fat tail distribution. This is why we gave lower weights to the separation based on the users' distributions, which could be sufficient by their significance. Rather, we added the bipartite separation to enable a more neutral inspection which is not based on the separation by users' distribution alone.

Following the reviewer`s comments, we better emphasized this issue as seen in the new paragraph added below:

Another issue and possible limitation that needs to be considered the fit of the distribution to the power law distribution. Some studies claim that the distribution of hashtags is indeed a Power Law Zhou et al. (2020), while others claim it is some generalized Zip's law Chen, Alexander, Oliveira, and Altmann (2020). While a true fit of many distributions to the power law is more seldom than expected (Clauset, Shalizi, & Newman, 2009), such exact fit is not critical in the practical aspect of our work. This is because the conceptual novelty of our work is the ability to differentiate between political and non-political cascades based on statistical patterns of repetitions or words and users. These patterns of repetitions are even correct if the distribution does not fit to a power law distribution but simply to any fat tailed distribution.

Comment: Fig 4 – What is the x-axis in graphs A and B? In all four graphs, there are more than six red points (which are described in the SI tables). The x-axis are the event index, where Disasters are shown first and political cascades second.

Answer: Thank you for this comment. Following the reviewer`s comments we adjusted the images to have this point clearer. we added also to the SI the full distribution of hashtags of Egypt and Iran that appear in Fig 4 but not in Fig 2.

Comment: Fig 5 The analysis on parts A and B does not need network analysis; they are the distribution of hashtags in tweets and tweets to hashtags, and B is the average number of hashtags.

Answer: Thank you for this comment. We realized this issue was not clear and improved it. Part A and B in Fig 5 show the degree distribution of the word-to-tweet network and not that of the hashtags or users alone. This network uses ALL the words in a tween and not only the Hashtags. Since political tweets might tend to use hashtags more often than nonpolitical users, we wanted to compare the entire appearance of words and not simply hashtags. For this we create the bipartite graph. The slope in Fig 5A is the slope of distribution of the degree distribution of nodes in the bipartite network, and not simply the hashtags or users' distribution. While strongly connected

Best wishes,
Dr. Alon Sela, on behalf of the authors.
