# Supplementary material for: Signals of propaganda—Detecting and estimating political influences in information spread in social networks
Source: PLoS One. 2025 Jan 30;20(1):e0309688. doi: 10.1371/journal.pone.0309688 (PMC11781619; doi:10.1371/journal.pone.0309688)
Supplement: Attachment — Submitted filename: Response to Reviewers.14.8.2024.docx [file pone.0309688.s010.docx]

PONE-D-23-25017R1

Signals of Propaganda - Detecting and Estimating Political Influences in Information Cascades in Social Networks

PLOS ONE

Dear Dr. Sela,

Thank you for submitting your manuscript to PLOS ONE. After careful consideration, we feel that it has merit but does not fully meet PLOS ONE’s publication criteria as it currently stands. Therefore, we invite you to submit a revised version of the manuscript that addresses the points raised during the review process.

Please submit your revised manuscript by Sep 09 2024 11:59PM. If you will need more time than this to complete your revisions, please reply to this message or contact the journal office at [plosone@plos.org](mailto:plosone@plos.org). When you're ready to submit your revision, log on to <https://www.editorialmanager.com/pone/> and select the 'Submissions Needing Revision' folder to locate your manuscript file.

Please include the following items when submitting your revised manuscript:

- A rebuttal letter that responds to each point raised by the academic editor and reviewer(s). You should upload this letter as a separate file labeled 'Response to Reviewers'.
- A marked-up copy of your manuscript that highlights changes made to the original version. You should upload this as a separate file labeled 'Revised Manuscript with Track Changes'.
- An unmarked version of your revised paper without tracked changes. You should upload this as a separate file labeled 'Manuscript'.

If you would like to make changes to your financial disclosure, please include your updated statement in your cover letter. Guidelines for resubmitting your figure files are available below the reviewer comments at the end of this letter.

If applicable, we recommend that you deposit your laboratory protocols in [protocols.io](http://protocols.io/) to enhance the reproducibility of your results. Protocols.io assigns your protocol its own identifier (DOI) so that it can be cited independently in the future. For instructions see: <https://journals.plos.org/plosone/s/submission-guidelines#loc-laboratory-protocols>. Additionally, PLOS ONE offers an option for publishing peer-reviewed Lab Protocol articles, which describe protocols hosted on [protocols.io](http://protocols.io/). Read more information on sharing protocols at <https://plos.org/protocols?utm_medium=editorial-email&utm_source=authorletters&utm_campaign=protocols>.

We look forward to receiving your revised manuscript.

Kind regards,

Gilad Ravid, Ph.D.
Academic Editor
PLOS ONE

# Response to reviewers

Journal Requirements:

Please review your reference list to ensure that it is complete and correct. If you have cited papers that have been retracted, please include the rationale for doing so in the manuscript text, or remove these references and replace them with relevant current references. Any changes to the reference list should be mentioned in the rebuttal letter that accompanies your revised manuscript. If you need to cite a retracted article, indicate the article’s retracted status in the References list and also include a citation and full reference for the retraction notice.

**Thank you for this important remark**. We have reviewed the full citations list and each citation in the article appears in the ref list and no more, and vise versa.

We removed the following citation:

*Reich, Peter B., Jacek Oleksyn, Ian J. Wright, Karl J. Niklas, Lars Hedin, and James J. Elser. "Evidence of a general 2/3-power law of scaling leaf nitrogen to phosphorus among major plant groups and biomes." Proceedings of the Royal Society B: Biological Sciences 277, no. 1683 (2010): 877-883.*

Mainly since it is too specific for the topic we show in this manuscript, and while it is related to power law in general, it is too far from the topic of this article. Thus, we thought its not relevant enough.

Additional Editor Comments:

The authors improved the paper and answered most of the reviewers' concerns. Reviewer 1 is satisfied with the results, but Reviewer 2 did not respond to requests to review the paper again.
Some minor issues still need to be resolved before we publish the paper.

**Thank you!** We will do our best to address these additional comments.

a. the paper positions itself as dealing with propaganda and cascade. As they appear in the paper, the two phrases are not used as their true meaning. As Twitter is a crowded, uncontrolled system, it is not precise to name information distribution as propaganda (neither good nor bad). This is even stronger when the authors examine hashtags, which, as they claim, are keywords that describe the tweet. Hence, if many use the same hashtag, it is not a sign of propaganda. For example, many use the hashtag "#YesWeCan" to indicate their support for Obama's campaign, but it is hard to call it a sign of propaganda.

**Thank you for thin important comment**. We debated a lot about this comment and summarize our answer below as well as inside the manuscript itself (Line 480-520).

Also, we removed the word cascade from the title of the work.

*Indeed, the line separating between propaganda, information bias and simple repetitions in twitter is a fine line. First, we will discuss the relations between information bias and propaganda, then discuss the role of authentic users in the spread of propaganda. We name in this work an information bias by any external force, aiming to bias the natural spread of ideas, as propaganda. This is a very broad definition, since it also includes commercial communication efforts to spread a name of a brand, as well as the messages from a charismatic opinion leader. This definition also includes the “darker” aspects of this phenomena such as the use of centrally or decentralized organized bots and fake accounts to spread misinformation.
What we see in our work is that although the existence of charismatic opinion leaders that repeat consistently some specific messages is for sure a natural phenomenon, (and cannot be considered as propaganda by itself), the distribution of such charismatic users and topics is more common in the political topics compared to the natural disaster’s topics.*

*This can be clearly observed in Fig 2, and Fig 3, where one can observe that the distribution slopes for the hashtags used in the political topics are less steep, thus, the decay in hashtags usage is slower in the political topics, representing higher repetitiveness in the language of the political topics.*

*In contrast, for the users, the slope is steeper for politics, thus some users are very active while the majority are barely active at all. These active users in the politics, that repeatedly spread the same hashtags again and again, and are thus the statistical footprint of propaganda. Furthermore, as long as we keep the neutral definition of propaganda, as a repetitive broadcast of a message, regardless if this is an acceptable or unacceptable message, we avoid the need for any evaluation of the good or bad moral aspect of the topic.*

*In this perspective, “Just do it” is a propaganda similarly to “From the river to the sea, Palestine will be free”, since we ignore our personal view of the content, and just inspect if the distribution of these topics is biased or unbiased.*

*Indeed, in general the term “propaganda” is used for a “bad” spread of ideas that includes a massive brainwash. We claim that any information bias is considered as propaganda, either if it’s for good or for bad reasons. Furthermore, while the reviewer`s observation that “yes we can” can be a legitimate message that has been spread by an authentic user, our “statistical” definitions of propaganda does not limit the term propaganda only to the direct effect between the government and the people. This is because such a narrow definition, will not define the burning of old scripts (either by German Nazy or by Young Mao enthusiasts), when no government gave a direct order to burn such books, as a natural phenomenon, but clearly as an act resulting of propaganda, even if such acts were not directly including the government, but only resulted in a crowd-to-crowd direct messages spread.*

*To conclude: in this work we define propaganda as any effort to bias the spread of information. This effort is generally hidden. Bots, fake accounts, smart slogans, are all a part or this effort, in which although most effects cannot be observed individually, our claim here is that they can be observed by their statistical properties.*

Line 36-49 Also, I need help finding a relationship between the users' power law distribution and propaganda.

**Thank you for this important comment that would help us clarify an important point**. The relationship between Power Law and propaganda passes through the positive feedback mechanism that in one of the processes that create power law. Propaganda is not an effective propaganda if it does not include the echoing of the message through the authentical audience. As mentioned in our previous work on this topic [ref], an effective way to spread a message is by echoing it in a recruited group, which we name “spreading group”, and then from this echoing, the message passes to the group of authentic users. We also name this spreading group as message “detonator” since they act to start the spread. This process, where authentic users are more likely to believe to a message thus tend to spread it themselves, is the positive feedback mechanism that can create a power law.

Furthermore, the data just shows that the hashtags and users are distributed according to a long tail distribution, this usually implies some dependence or positive feedback in the process, compared to an independent process which create a normal distribution.

We define “Propaganda” as a repetition of a same idea over and over. Under this definition, also the **yes-we-can** hashtag is considered as propaganda. This is even more true if “regular” people start using this term as a result of the initial mention of it by politicians, thus echoing it further more. Such initial echoing of a message, starts by the “Spreading Group” but then, later spreads through naïve users. We have found already that it exists in groups of humans and bots in stock markets groups.

As regards the cascade term, the research does not look at the cascade of information; cascade happens when someone transfers ideas or information, he hears from someone else. There is no indication of transferability in the study.

**Thank you for this comment.**

It is important to note that all information cascades in twitter lacks the full link and information path. This is because when user (A) retweet user (B), and then user (C) retweet user (B), it will appear in the Twitter as user (C) retweeted (A). Nevertheless, these information spreads are generally named cascades, although in practice we are never sure what was the path of the true cascade and what is an artifact.

Indeed, one can only use retweets, which are a stronger indication of a cascade. We added to the discussion section a short paragraph mentioning this real limitation.

The authors use the term "political cascade" to denote the political networks. Political can cascade information, ideas, and knowledge but can't cascade politics.

**Thank you for this comment**: we clarified the term:

Line 242: should be a curve fit line (you miss the "v"). **Thank you** – corrected!

Line 252 vs. line 267: Yi is index or frequency (I think the letter).imilarly in both places. Y(i) is the frequency of the appearances of each hashtag. **Thank you**! we corrected this error in the revised manuscript.

**Thank you for this comment**. We redefined Y(i) correctly Line 273:

Please use the standard notation in the equation; the predicted value is Y hat (^), and the upper bar is reserved for the mean.
**Thank you!** Corrected.

Line 280: both sides of the equations are exactly the same.

**Thank you for this comment**. We corrected the equation. We did not color this change in red, due to it being in a formula.

Throughout the article and equations, the authors interchangeably treat alpha as slop and alpha as minus the slop. For example, line 333 states that alpha equals -763 (you miss the decimal point. It should be -0.763), but on line 276 the line equation considers alpha as positive (the minus is part of the equation)
**Thank you for this correction**. We kept consistency and mentioned all alpha values as positive, while when mentioning the slope, we mention the negative number.

Please add comments and refer to some prior works on the differences and problems of fitting power law directly vs. fitting to the log-log of the values. Goldstein, M. L., Morris, S. A., & Yen, G. G. (2004). Problems with fitting to the power-law distribution. The European Physical Journal B-Condensed Matter and Complex Systems, 41, 255-258. is one candidate reference ( the problem related to the changing distribution of the log(error) you introduce)

**Thank you for this comment**. Ref Added!

line 254 vs. line 312: Did you make least square error fitting or maximum likelihood fitting?

**Thank you for this important comment!**

We used logarithmic binning and least square methods after binning on a logarithmic scale. While some researchers claim that Maximum Likelihood method (that is strongly advocated by Clauset et al) is a best method to estimate the Power Law slope, such that it will not to get a biased slope due to the long tail, other recent research, after generating power law distributions and seeking their (known) slopes, claim otherwise. This method ensures removing the bias toward an under-estimation of the slope due to the long tail, and was chosen following a [recommendation of Prof. S. Havlin](https://scholar.google.com/citations?hl=en&user=mB6GO8UAAAAJ&inst=9485470196299243772) (h-index 150), a world known expert in this topic.

Also, the work of (Zhong, 2022) has investigated this issue, and has concluded that *“…the criticism about the inaccuracy of LSE in fitting power-law distributions is complete nonsense” ,…* and also that *“. Our experiments uncover a fundamental flaw in the widely known CSN2009 method proposed by Clauset et al. [12]: it tends to discard the majority of power-law data and fit the long-tailed noises. Such flaw invalidates the reliability of all the research based on CSN2009 and all those works (e.g., [10, 21, 39, 52, 54]) need to be re-investigated”*

Furthermore, the logarithmic binning that we have performed, overpasses the problem of the long tail from one hand, and the higher points density in the low values from the other. It has been shown (Milojević 2010) that when the data is **not a clean Power Law**, but rather an approximation of the power law, then a logarithmic binning will result in an unbiased slope estimate using LSE methods, and will perform better than ML if the distribution is not a pure power law (as in our case).

Along this line, we also tried using the method of Maximum Likelihood method, but it did not result in slopes that fitted our data visually, and since we could observe the slopes differences visually, we preferred to stick to slope measuring method by logarithmic binning and LSE that better fit the visual slope and its seen differences between the political and non-political discussions.

Milojević, Staša. "Power law distributions in information science: Making the case for logarithmic binning." *Journal of the American Society for Information Science and Technology* 61, no. 12 (2010): 2417-2425.

Zhong, X., Wang, M. and Zhang, H., 2022, April. Is least-squares inaccurate in fitting power-law distributions? The criticism is complete nonsense. In *Proceedings of the ACM Web Conference 2022* (pp. 2748-2758

Line 306: In your answers to the reviewers, you stated that you didn't perform betweenness calculations; in the paper, you did.

**Thank you for this comment**. The betweenness was computed for the bipartite graph. It was not one of the most effective separator thus we did not continue using it.

Line 337 vs. Fig 2, Bangladesh 2019 slope is 0.976.

**Thank you very much**! We corrected this err!

Line 340 vs. caption of Fig 2. What is the mean 0.97 or 0.938
**Thank you for this remark.** We improved the caption to explain this difference which arise from the mean of the 6 shown in the image (-0.938) and the mean of the 10 cascades (-0.973).

[Note: HTML markup is below. Please do not edit.]

Reviewers' comments:

Reviewer's Responses to Questions

**Comments to the Author**

1. If the authors have adequately addressed your comments raised in a previous round of review and you feel that this manuscript is now acceptable for publication, you may indicate that here to bypass the “Comments to the Author” section, enter your conflict of interest statement in the “Confidential to Editor” section, and submit your "Accept" recommendation.

Reviewer #1: All comments have been addressed

**Thank you for this agreement!**

2. Is the manuscript technically sound, and do the data support the conclusions?

The manuscript must describe a technically sound piece of scientific research with data that supports the conclusions. Experiments must have been conducted rigorously, with appropriate controls, replication, and sample sizes. The conclusions must be drawn appropriately based on the data presented.

Reviewer #1: Yes

3. Has the statistical analysis been performed appropriately and rigorously?

Reviewer #1: Yes

4. Have the authors made all data underlying the findings in their manuscript fully available?

The [PLOS Data policy](http://www.plosone.org/static/policies.action#sharing) requires authors to make all data underlying the findings described in their manuscript fully available without restriction, with rare exception (please refer to the Data Availability Statement in the manuscript PDF file). The data should be provided as part of the manuscript or its supporting information, or deposited to a public repository. For example, in addition to summary statistics, the data points behind means, medians and variance measures should be available. If there are restrictions on publicly sharing data—e.g. participant privacy or use of data from a third party—those must be specified.

Reviewer #1: Yes

5. Is the manuscript presented in an intelligible fashion and written in standard English?

PLOS ONE does not copyedit accepted manuscripts, so the language in submitted articles must be clear, correct, and unambiguous. Any typographical or grammatical errors should be corrected at revision, so please note any specific errors here.

Reviewer #1: (No Response)

6. Review Comments to the Author

Please use the space provided to explain your answers to the questions above. You may also include additional comments for the author, including concerns about dual publication, research ethics, or publication ethics. (Please upload your review as an attachment if it exceeds 20,000 characters)

Reviewer #1: The authors have addressed my concerns.

Thank you for this positive feedback!!

In particular they added a section on their method of PLMSE better explaining the model. They also added statistical tests that are more convincing for the segregation between the datasets.

7. PLOS authors have the option to publish the peer review history of their article ([what does this mean?](https://journals.plos.org/plosone/s/editorial-and-peer-review-process#loc-peer-review-history)). If published, this will include your full peer review and any attached files.


If you choose “no”, your identity will remain anonymous but your review may still be made public.


**Do you want your identity to be public for this peer review?** For information about this choice, including consent withdrawal, please see our [Privacy Policy](https://www.plos.org/privacy-policy).

Reviewer #1: No
